# Current challenges and future directions for naturopathic medicine in Australia: a qualitative examination of perceptions and experiences from grassroots practice

**DOI:** 10.1186/1472-6882-13-15

**Published:** 2013-01-14

**Authors:** Jon Lee Wardle, Jon Adams, Chi-Wai Lui, Amie Elizabeth Steel

**Affiliations:** 1 Faculty of Health, University of Technology Sydney, 235-253 Jones St, 2007, Ultimo, NSW, Australia; 2 School of Population Health, University of Queensland, Herston Rd, 4006, Herston, Qld, Australia

**Keywords:** Naturopathy, Regulation, Professional issues, Practitioner perceptions

## Abstract

**Background:**

Naturopaths are an increasingly significant part of the healthcare sector in Australia, yet despite their significant role there has been little research on this practitioner group. Currently the naturopathic profession in Australia is undergoing a period of rapid professional growth and change. However, to date most research exploring the perceptions of naturopaths has been descriptive in nature and has focused on those in leadership positions rather than grassroots practitioners. This article explores the perceptions and experiences of practising naturopaths on the challenges and future directions of their profession.

**Methods:**

Semi-structured interviews were conducted with 20 naturopaths practising in the Darling Downs region of South-east Queensland, Australia to explore current perceived challenges in the naturopathic profession in Australia.

**Results:**

Participants perceived a number of internal and external challenges relating to the profession of naturopathic medicine. These included a public misconception of the role of naturopathic medicine; the co-option of naturopathic medicine by untrained or unqualified practitioners; the devaluation of naturopathic philosophy as a core component of naturopathic practice; a pressure to move towards an evidence-based medicine model focused on product prescription; the increasing commercial interest infiltrating complementary medicine, and; division and fragmentation within the naturopathic profession. Naturopaths generally perceived government regulation as a solution for many of these challenges, though this may be representative of deeper frustrations and disconnections between the views of grassroots naturopaths and those in professional leadership positions.

**Conclusions:**

Grassroots naturopaths identify a number of challenges that may have significant impacts on the quality, effectiveness and safety of naturopathic care. Given the significant role naturopaths play in healthcare in Australia the practice and policy implications of these challenges require further research attention.

## Background

Complementary and alternative medicine (CAM) practitioner consultations may account for half of all health consultations and half of all out-of-pocket healthcare costs in Australia [1]. Naturopaths are the largest and fastest-growing CAM practitioner group in Australia [2]. Naturopathic medicine defines itself as a system of primary health care: an art, science, philosophy, and practice of diagnosis, treatment, and prevention of illness. Naturopathic medicine is not defined by the substances used but rather by the principles that underlie and determine its practice, which include the following: supporting the healing power of nature, finding the root cause of ill health, first doing no harm, treating the whole person, prevention, and doctor as teacher [3,4].

The use of naturopaths by the Australian public is high, with large longitudinal studies indicating over 10% of middle-aged women consult with naturopathic practitioners [5], increasing to over 15% in chronic, complex or serious conditions such as cancer [6]. In many cases naturopaths are the primary care providers for Australian patients utilising their services [7-9], and naturopaths are now the largest unregulated health profession in the country with a major primary care role [10].

However, unlike many other CAM professions in Australia, the profession of naturopathic medicine remains significantly fragmented, with over 90 associations purporting to represent Australian naturopaths [11], which in addition to compounding the heterogeneity of practice and education standards [12] has also hampered professional development due to professional infighting [11,13-15].

Despite the growth in naturopathic practitioner ranks and high utilisation of naturopathic services in Australia, little research has been focused on this practitioner group. Existing studies on this topic have been primarily descriptive in nature, focusing on practitioner profiles and demographics [11,16], the practice environment [9,17-23], and education, training or regulatory developments [12,14,24-26].

Early exploratory qualitative work has explored perceptions and opinions of Australian naturopaths on major professional issues such as regulation [27]. However, this study was confined to researching those occupying leadership roles in the profession. More broadly, commentaries on issues affecting naturopathy in Australia seem to be dominated by the professional ‘elites’ with the perspectives of grassroots practitioners underrepresented [10]. Even in the United States and Canada where the naturopathic communities have been more intensively studied, such research tends to be descriptive and has seldom explored professional and practice issues at a deeper level, and have also focused on the opinions of those in leadership positions [28,29].

In response, this paper aims to remedy this gap in the evidence base by providing an analysis of the perceptions and experiences of grassroots Australian naturopaths regarding the current challenges and future direction of their profession. The study provides a crucial first step in understanding the perceptions and beliefs of grassroots practitioners.

## Methods

Qualitative semi-structured interviews were chosen for data collection to explore the perceptions and experiences of grass-roots naturopaths regarding their profession [30]. Ethical approval for the study was obtained from the School of Population Health Research Ethics Committee, University of Queensland in accordance with the guidelines set by the National Health and Medical Research Council.

Study participants were drawn from naturopaths in current practice in the Darling Downs region of South East Queensland. All naturopaths in that area who were registered with any one of Australia’s four largest accrediting professional associations for naturopaths (Australian Natural Therapist’s Association; Australian Naturopathic Practitioners Association; Australian Traditional Medicine Society; and National Herbalist’s Association of Australia) were contacted and invited to participate in the study. The researchers contacted all practitioners (31 in total) in the area and all those who expressed interest in the research were interviewed. A total of 20 interviews were conducted. Table 1 provides an overview of characteristics of the participants and compares them with data from the general naturopath population based on the latest workforce survey [10].

**Table 1:** Characteristics of the study participants compared to the general naturopath population in Australia

	**Participants**	General naturopathic population*
Female	55%	76%
Average Age	39	44
Average (Formal) Training Length	4 years	3 years
Average Years in Practice	8 years	7 years
Average Weekly Hours in Clinic	32 hrs	25 hrs

* Source: Lin, V. et al. *The Practice and Regulatory Requirements of Naturopathy and Western Herbal Medicine*. Melbourne: Department of Human Services, 2005.

Interviews were conducted at a place and time convenient for participants. Written informed consent was obtained from participants prior to interviews being conducted. The interviews were taped and were an average of 60 minutes in length. A theme list of questions was prepared to guide the interview, but the participants were encouraged to shape the discussion in line with their own perspective, focus and concerns. Keywords, phrases and arguments used by the naturopath were noted and their meanings clarified as the interview proceeded.

All tapes were transcribed verbatim shortly following interview. Data analysis was undertaken concurrent to the processes of data collection with codes and analytical themes developed in a cumulative manner. To enhance validity, the research team coded the transcripts separately and compared the results afterward. The resultant codes and themes were then fed back into subsequent coding.

## Results

Data analysis identified a number of concerns among the naturopaths regarding current challenges facing the naturopathic profession.

### Misconception and erosion of naturopathic philosophy

When asked to highlight strengths of their profession, most participants emphasised that the effectiveness of naturopathic care was related less to the use of specific products and more to the underlying principles and guiding philosophy of naturopathic practice. It is noteworthy that even though the individual treatments used by practitioners were quite heterogeneous amongst participants, there was a general consensus among the participants on basic principles of naturopathy that are consistent with international definitions [3,4]. All participants identified these underlying principles as being the “true essence” and defining factors of what it was to be a naturopath rather than being defined by their tools of trade (such as the dispensing of complementary medicines). As the following quote illustrates, adherence to these principles was seen by the participants as the core tenet differentiating naturopathic practice from other therapeutic disciplines.

“You can get supplements anywhere… but you can’t put a… personal… program… or a personal herb mix together without that particular [naturopathic] knowledge… and I think that is a really important part… it’s that individual prescribing and those core principles… it can’t all be scientific… it’s just often left-brained” (PE)

Respondents perceived growing pressure on naturopaths to base clinical decision making entirely on biomedical model of diagnosis and “disease-naming”, which was a matter of concern amongst most respondents. Movement towards what practitioners described as “green allopathy” or “medicalised naturopathy” was seen by many participants as an anathema of the basic principles of naturopathy, which held grave consequences for naturopathic practice, as the quote below demonstrates:

“I remember training a few students in the student clinic and a couple of them came up to me and said this patient… she had all these poxy sores on her legs… and they said… the doctor told her she had… I can’t remember what it was… and we haven’t been taught how to treat that… and I said it’s irrelevant… go back to your basic principles… the skin is an organ of elimination… you’ve told me here that the bowel function, the kidney function is poor… activate that and the load will disappear off her skin… they did that and thought that was revolutionary… she came back about three weeks later and her sores had cleared up about 60-70%… but we’re losing the basic thrust of what we do by trying to integrate into that medical system of just treating a disease” (CT)

However, despite reservations on biomedical model dominance in the profession, most respondents held little objection to raising the level of biomedical knowledge and training amongst practitioners, as long as the basic principles of naturopathy were upheld:

“Oh yes… proper science training is absolutely essential… but so is proper naturopathic training… science… health science… is the common language of medicine… you can’t be any kind of practitioner without it… but you can’t be a naturopath without knowing the other side properly either” (BN)

Many respondents were also open to the idea of further integration of naturopathic and biomedical knowledge and supported the teaching of increased levels of biomedical sciences in naturopathic education, provided that this was not at the expense of the philosophy of naturopathy. As illustrated in the following quote, whilst some participants were supportive of integration with ‘scientific’ medicine, they rejected co-option or usurpation:

“If we’re going to go in the more scientific aspect we could lose that individual sense about it… if we become too far attached to the scientific establishment we may be being asked to put up barriers… we might get that part taken away… and we could end up losing what actually makes us naturopaths… what makes us different” (JS)

### Pressure to move towards an evidence-based paradigm

Although generally supportive of the need for increased evidence for their therapies, many respondents expressed concerns with what they perceived as the pressure to take up what they viewed as a “dogmatic” evidence-based medicine (EBM) approach in naturopathic treatment. In particular, they feared that the EBM approach may downplay a traditional emphasis on treating patients individually as well as drawing upon the “art” of the practitioner. As can be observed in the quote below, some respondents even suggested that the introduction of an evidence-based approach to their practice may harm the profession more generally:

“I think a lot of the industry is trying to justify itself by going down the medical research model… and I think that’s very foolish… I think that we’re too busy trying to play the doctor’s games… we’re having to play on their turf… all that matters to me is that someone sees me and they get better… that’s all the evidence I need… but they’re using that lack of evidence as an excuse to close us out… but it’s their kind of evidence… and it doesn’t work that way for us” (DH)

Participant concern on the impact of an evidence-based approach was not confined to the area of practice. Many participants also complained of the trend of contemporary naturopathic education to “become more scientific”, primarily so that the discipline can be “accepted in the university sector”, and claimed that such a development would be undertaken at the expense of the philosophical underpinnings of the profession.

### The commercialisation of CAM

Participants also expressed concern with the perceived growing commercialisation of CAM. In particular, participants feared that naturopaths experienced growing pressures to “push product” instead of treating patients. Participants highlighted that there was a significant ‘product pushing’ practitioner component within their profession and some participants admitted to experiencing personal conflicts in their roles as prescribers and sellers of therapeutic products in their clinics.

Respondents expressed concern that the increasing influence of therapeutic product manufacturers – and more specifically their protocols – risked “overriding the art of being a naturopath”, and forcing naturopaths to move increasingly towards specific therapeutic product prescription rather than devising individual treatment plans for patients. As one naturopath noted:

“The pharmaceutical companies – and that’s what they are now – have so much influence over practitioners… you used to treat individually but now they say ‘Oh it’s a prostate problem, use [specific commercial prostate product] or whatever’… it’s like we’ve just become sales reps for them… and now they’re providing most of the education… it’s a bit worrying really” (DH)

Many participants viewed product prescription and evidence-based practice as wholly similar concepts, as evidence was generally perceived to be largely focused on individual products and not individualised therapeutic treatments or non-product therapeutic modalities. Increasing commercial influence from the product sector, as perceived by participants, was seen to have intensified the continued undermining of naturopathic clinical experience by company’s promotion of practice centred upon product prescription as “evidence-based” practice. The following quote summarises well this concern of the respondents:

“I think the medical herbalism model has done untold damage to the profession… because basically it’s making a [conventional] medical diagnosis and using these… drugs… to treat symptomatically… and it’s not looking at the emotional side… psychological state… a host of other things… just [ignoring] basic naturopathic principles” (MS)

However, this move toward a prescription-oriented practice was also perceived by some participants to be in part demand driven by patients. Many practitioners suggested that they had often felt pressured by patients to “get them to leave with something”, with patients often preferring ‘medicine’ to ‘treatment’ and “wanted to take something home with them” after each consultation.

### Division and fragmentation within the naturopathic profession

Participants also identified significant challenges facing their profession and arising from *within* the ranks of their profession. Naturopathy was perceived as a divided and fragmented profession, with casual comments on divisions and lack of collegiality amongst practitioners expressed by all participants. Additionally, most participants regarded such internal divisions and disagreements as a core challenge facing their profession. It was suggested by one respondent (HS) that “the naturopathic community doesn’t need any enemies, it’s too busy fighting with itself”. As illustrated in the quote below, some participants believed the profession’s focus on challenges from the conventional sector were unwarranted:

“You know… you hear all this talk about how the medical profession is out to get us but from my experience it’s simply not true… there is no real ill will from them at all… in my opinion naturopaths have been holding themselves back more than anyone else has… you look at the associations and the leaders in the profession and there’s so much infighting… hostility… some groups can’t even stand being in the same room.... and that’s caused more damage than anything else… really the profession needs to get its act together on that front or it’s never going to get anywhere” (BD)

Some participants suggested that internal divisions may have their roots at the college level, recounting how as naturopathic students they seldom interacted with other disciplines and rarely interacted even with naturopathic students from other institutions. Although some participants suggested this lack of interaction was purely circumstantial and accidental, other participants suggested a more strategic and antagonistic approach was fostered by college heads and administrators, who often viewed competing colleges as “the enemy”. This adversarial attitude was seen as extending beyond the education sector, with many participants highlighting how market realities lead to unhealthy competition and rivalry rather than collegiality between local practitioners. As the quote below illustrates, this competitive practice environment was seen by some participants as a serious obstacle to developing collaboration and support networks amongst practitioner ranks:

“It’s pretty competitive… you talk to a naturopath and they talk about how busy they are… how many patients they’re seeing but not much else… they don’t want to talk too much in case you… I don’t know… might steal their patients” (WX)

Participants also explained how divisions within the discipline could render professional naturopathic practice a lonely experience, with many participants highlighting uncertainty regarding where, or with whom, they could discuss professional issues. As the following quote demonstrates, participants thought this professional isolation could affect their practice:

“It would be nice to have some kind of support with all that stuff [professional issues]… sometimes you have a really bad day or you’re worrying about the business and you don’t get to devote yourself to your patients… I think they can tell sometimes” (JC)

Some participants perceived that lack of adequate professional leadership and resultant fragmentation deprived the naturopathic profession the opportunity to control its destiny. For example, participants discussed how the product manufacturers had filled the vacuum left by associations and colleges to become major providers of professional education and support for practitioners. As a result, it was perceived that practitioners were unable to discuss professional or technical issues with independent or professional sources, and that advice sought in this manner quite often ignored naturopathic principles and focused instead on reductionist or protocol based product prescription. This often left participants feeling conflicted, needing to balance their desire for professional support with ensuring that they are not swayed by any conflicts of interest, as can be observed in the following quote:

“There really is no-one up here to talk to… all my friends that I studied with… they’re in Melbourne… Sydney… the associations don’t really do a lot… the [supplement company] practitioner hotlines are pretty helpful… I use them quite a lot… but sometimes you’ve got to wonder if it’s ethical taking advice from those people… surely it’s a conflict of interest” (KJ)

### Co-option of the naturopathic title by unqualified persons

One of the prescient issues for naturopaths in the study was the confusion amongst those outside the profession as to precisely what a naturopath actually is or does. As two participants explained:

“I think it’s just education [that’s needed] really… we need to show people that we’re not just going around shaking chickens over people’s heads and chanting… we’re not just crazy hippies… no-one knows what we do or what we’re about and that’s one of the major problems” (JS)

“It concerns me a little bit… sometimes spiritual healers or obscure types of treatments get seen as quackery and they’re taking away from the profession…I mean people get a bad experience and you get lumped in with them… no one knows what we do – people go ‘oh you guys do reiki… kinesiology’… you know there’s a place for these medicines but you need that extra training to be a naturopath… these guys [non-naturopathic CAM practitioners] give the profession a bad name because they’re not naturopaths and everyone thinks that they are” (MS)

Some participants perceived that the public’s confusion over naturopathic training and scope of practice, combined with an unregulated practice environment and co-option of the term “naturopath” by unqualified persons that participants described as “quacks”, “charlatans” and “shonks”, helped to reinforce the conception that naturopathy was not a legitimate or scientific practice. Participants discussed the frustration they felt at the “unscientific” labels often branded upon naturopaths and their practice, when they themselves thought that scientific training was integral to naturopathic practice. As one practitioner explained:

“Most [conventional medical practitioners] are surprised at how scientific the whole practice is… I tell them exactly what is happening and what this or that will do to them… they really are quite surprised at my knowledge… not that my knowledge is anything special… well I guess it is but not as far as naturopaths are concerned… I think we should all be expected to know those things” (KJ)

This confusion, with the common co-option of the naturopathic identity by other less-trained practitioners wishing to be conferred “higher status”, was perceived by some interviewees as devaluing the naturopathic reputation in Australia. Regulation of the profession was seen by many practitioners as the only way that this challenge could be overcome. As one naturopath explained:

“Registration will fix everything… otherwise problems will multiply… people will walk into health food stores and expect to get free advice and the prescription products… there’ll be more and more bad stories on A Current Affair… people get the wrong advice and then they blame us and it’s never us that say it… it’s the people pretending to be naturopaths… not the actual naturopaths themselves” (MC)

### Proposed solutions to current challenges facing naturopathy

Participants proffered perceived solutions to the challenges facing naturopathy in Australia. One such solution, as seen in the previous quote, was regulation of the profession, and was in fact seen by many participants as the core solution to many of the profession’s problems. Only one participant expressed a negative attitude towards regulation, though offered this opinion in frustration– stating “it wouldn’t change anything anyway” - rather than being indicative of in-principle opposition to the concept.

For many participants regulation was held up to be a panacea for many of the problems affecting the naturopathic profession – including the growth of external influences (“minimum standards will hold them accountable”) and the fragmentation and division within the profession (“what will they have left to fight about?”). Regulation in this sense was used by many practitioners as a comprehensive all-inclusive term of convenience that could also be used to discuss other issues generally considered to be directly related to regulation, such as professionalization, acceptance by the conventional healthcare system and problems in naturopathic education.

However, the primary reason offered by participants for this support for professional regulation was the potential to rid the profession of those practitioners participants deemed unethical, bogus, or fraudulent which were perceived by participants to be co-opting or “hijacking” the title of naturopath without the pre-requisite qualifications, which ultimately devalued the naturopathic brand. As outlined in the quote below, some participants believed that this devaluation made it more difficult for “true” naturopathic practitioners to integrate or communicate with conventional providers, who often have experience with these rogue practitioners:

“I’ve had a couple of doctors ask me about what I do… which is great… and they’re always pleasantly surprised after talking to me… but then they tell me stories of other experiences they’ve had with other naturopaths… like one doctor was telling me this story about a guy that saw a naturopath and he just wanted to hold him upside down during the treatment… and I just thought… Christ… is that what you think we do… they have no idea really” (WX)

The issue of bogus practitioners highlighted an acknowledgement by participants that there were problems within the profession. However, despite perceiving numerous challenges related to these elements, most participants anticipated a bright future for naturopathy in Australia, but one that could only manifest if underlying issues such as the “weeding out” of the profession’s “dodgy” element could be resolved. As one respondent explained:

“I think that naturopathy is going to be bigger than most people expect… with this global push for better health we could be one of the key things to sorting out the system… but we have to sort our stuff out… there’s a lot of loose ends in the industry and we need to… I’d love to cut the dodgy [practitioners] off… they’re the ones hurting my practice at the moment” (LS)

## Discussion

Naturopaths expressed concerns about the perceived co-option of their professional title, and the devaluation of their profession this enabled, which was somewhat assisted by the fragmentation of their profession. The concerns exhibited by naturopaths in this study, particularly around the loss of the underlying principles and philosophies by which the practitioners define themselves, do not seem isolated to naturopaths, and mirror concerns expressed by CAM therapists in Australia more generally [31].

However, findings from this study also highlighted the difficulties practitioners have in enabling the public to make distinctions between naturopathic practice and other forms of complementary health care. Although there was consensus amongst participants about specific naturopathic theory, and they acknowledged that this was re-enforced through every aspect of comprehensive naturopathic training, they acknowledged that the lack of public awareness of naturopathic theory and philosophy as an essential element of practice made it difficult to defend the tenets of the profession. This also appears to be an issue in the profession internationally, and through initiatives such as the Foundations of Naturopathic Medicine project the profession has recently undertaken significant efforts to codify naturopathic theory and philosophy to address these definitional issues [32]. However, the unregulated nature of the naturopathic profession in Australia perhaps makes the professional implications of this issue (e.g. co-option by external forces or the nefarious influence of untrained practitioners) more acute [14]. In the absence of this core foundational support from professional institutions, it is therefore perhaps unsurprising that the practitioners in this study looked to external regulation to define their roles in the Australian health care system. The naturopathic ‘art’ of practice, although highly regarded by participants and potentially containing unique benefits, remains largely unexplored. Given the prominence practitioners place on the specific aspects of the naturopathic approach, further research exploring these specific aspects of the naturopathic approach and identifying what value they may have in health care delivery is warranted.

Regulation formed an integral part of the solution for many of the professional challenges perceived by naturopaths in this study. The belief of practitioners that regulation “would fix” many of the problems within the profession, even those not necessarily related to professional or practice standards (such as uniting the profession), is a finding consistent with quantitative research that suggests that support for statutory regulation amongst the naturopathic workforce is related to a variety of issues, not just increasing professional standards [10,11,33].

However, the high level of support for professional regulation exhibited by grassroots practitioners in this and previous studies may directly conflict with opinions held by naturopaths occupying leadership roles within the profession. Previous qualitative exploration of naturopaths with senior roles in professional associations in Australia suggests that these practitioners exhibit a negative attitude towards statutory regulation [27]. Additionally, the concerns of erosion of naturopathic principles and philosophy also do not seem to be shared by naturopaths in professional leadership. For example, in some instances, professional associations are actively promoting the co-option of naturopathic medicine by broader natural medicine practitioners, who may not have philosophically-based naturopathic training [34,35].

Divergent views on issues considered important by grass-roots practitioners in this study and those in professional leadership roles may be an underlying factor behind the professional isolation and frustrations felt by naturopaths in this study, and deserve closer attention. Legislation which mandates professional association membership may also mean that professional associations are not compelled to represent practitioner interests.

Divergent views between grassroots naturopathic practitioners and naturopaths with leadership roles in the profession may also be suggestive of generational differences between the two groups. In her exploratory investigation of professional leaders in naturopathy’s attitudes to regulation, Canaway noted significant differences in post-1996 and pre-1996 graduates [27]. Most naturopaths in current practice have graduated since 1996, the year naturopathic training was first offered at a degree level in public universities, whilst naturopaths who have dominated professional leadership positions predominantly graduated before this time, usually from individually-owned smaller proprietary colleges [12]. Post-1996 graduates are more likely to have received extensive biomedical and scientific training, which results in a less adversarial approach to working with the conventional health sector [26,27].

Pre-1996 graduates, on the other hand, were trained in times when naturopathy was seen as unconventional, dangerous, and – in one government report – even derided as “a minor cult system” [36]. Older naturopathic graduates may have stronger resistance to higher levels of science and biomedical training in university and degree courses, which they may view as degrading naturopathic principles [24], and many in leadership roles may consider themselves the protectors of ‘sacred’ naturopathic principles and philosophies, even though these views appear to be representative of ‘natural medicine’ rather than naturopathy [27].

A simpler explanation of the apparent discrepancies between the perspectives of naturopathic ‘leaders’ (as identified in other work) and those of grassroots practitioners (as identified in our study) is that those in naturopathic leadership roles, have different interests than grassroots practitioners, and therefore do not share the interests of the broader practitioner base. Commentators have suggested that protection of financial self-interest through college ownership, or protecting the political power of professional associations by controlling registration and accreditation of practitioners are often the primary reasons for those in professional leadership roles to resist regulation [12-15,33].

Although the discrepancy between grassroots and professional leadership opinion is particularly evident in the regulation debate – which seems the most heated contemporary debate among naturopaths in Australia [27] – it may be present in many professional issues. Many practitioners interviewed explained how they felt unrepresented or left without any support within their profession, many lamenting the lack of a unified voice representing their interests, and some even going as far as to suggest internal politics had “hijacked” or “was destroying” their profession. Practitioners in this study seemed resigned to not being represented by their professional representatives, and therefore support for regulation may be totemic of support for a newer, and more inclusive, professional hierarchy within the profession.

In addition to concerns on professional issues, practitioners in this study also identified challenges that were more concerned with clinical practice. Respondents expressed concern at the growing influence of manufactured CAM product in their practice. The phenomenon of CAM being increasingly recognised as a commercial healthcare ‘product’ have been raised previously by commentators [37], as have the potential conflicts of interest of product sales by CAM clinicians [22,38]. The cautious views of naturopaths in this study on this issue seem not only consistent with external critics of the CAM industry, but also with CAM practitioner views elicited in other studies, which have highlighted practitioner concerns with the increasing commercialisation of CAM, with practitioners expressing concerns that the increase in prevalence of commoditised form of CAM may force practitioners to take a ‘business-like’ attitude to CAM, often at the expense of an altruistic focus on patients [31].

The shared concerns of CAM industry critics and CAM clinicians in this study may suggest that the emerging ‘product focus’ may be supply, rather than demand driven, at least from the perspective of naturopaths. Respondents observed that in practice they are under pressure from patients to prescribe or dispense specific kinds of CAM products, and patients’ expectation for prescription may be a product of aggressive marketing of CAM manufacturers [39]. However, most respondents rejected the notion of products alone as the formative tools of naturopathic practice, suggesting it was not the tools they used, but ‘how they used them’ that made naturopathic practice. Participants often considered the status of ‘product pushers’ as derisively as the ‘shonks’ and ‘charlatans’, and rejected the notion that they were simply the dispensers of natural medicines. Despite the perceived value of practice over product, Australian data suggests that most naturopaths do consider dispensing an important part of their profession, with 98% of naturopaths dispensing CAM products in their clinic [18].

Concerns over ‘product-focused’ approaches to healthcare delivery by naturopaths in this study may also partly explain the resistance to the EBM model of practice by some practitioners, who often perceived the ‘EBM approach’ as being synonymous with product prescription, as ‘acceptable’ evidence was usually in the form product trails. In this sense many naturopathic practitioners perceived EBM as supporting this shift away from the ‘art’ of naturopathic practice towards the ‘scientific approach’ which was dominated by product prescription.

These concerns echo those realised by both conventional and CAM practitioners in previous studies [40-44], and the self-perceived complex and holistic ‘practice-focused’ nature of naturopathy, which was not seen to align well with reductionist methodologies, amongst participants in this study may explain why some commentators have suggested that CAM and EBM are “divergent philosophical approaches” [45].

Although the naturopaths in this study highlight concerns about uncritical acceptance of a dogmatic EBM model and the negative effects this could have on naturopathic practice, they also incorporated a broadly positive view of an increasingly scientific approach to naturopathic training and practice, which incorporates increased biomedical training. These findings seems consistent with previous studies of naturopathic perspectives of science and evidence, which demonstrate naturopaths exhibit a complex and critical approach to evaluating and incorporating scientific and evidence-based perspectives in practice [21,46]. This perceived separation or distinction between EBM and biomedical science appears somewhat divorced from current perceptions portrayed in the conventional medical literature that reluctance to adopt an EBM model on the part of CAM practitioners is entirely supportive of the ‘anti-science’ and ‘risky’ element of this medicine [47]. In fact, some commentators have highlighted that there are numerous opportunities in EBM for naturopathy, but that evaluation simply requires the appropriate evidentiary tools, many of which already exist in numerous underutilised conventional health research methodological approaches [48,49].

What seems more evident amongst naturopathic practitioners in this study is a desire for a critical approach to the application of evidence and biomedicine to naturopathic practice – one that enriches naturopathic practice rather than replaces it. Increased research and a larger evidence base is a goal that naturopathic practitioners seem amenable to, though they desire the development of an evidence-base that accurately reflects their practice rather than one that is imposed and ignores the underlying philosophies that define their health care approach. These goals can be observed in the professions attempts to build research capacity and develop an international research agenda for naturopathic medicine, and their attempts to embrace this development as a necessary foundation for the future development of the profession [50].

As an exploratory study, this research draws upon a self-selected sample of naturopathic practitioners in one region of Australia. The use of a self-selected sample may limit the generalisability of the respondents’ observation on the respective practice, particularly when considering the variance created by the unregulated nature of the naturopathic profession in Australia. More research, both qualitative and quantitative, is needed to corroborate the findings of this study.

Given the significant role that naturopaths play in healthcare delivery in Australia, it is imperative that further research is conducted on naturopaths and their practice. Considering the significant differences that have been observed between grassroots practitioners and those in naturopathic leadership positions, it is essential that any research on this community be more inclusive of practitioners ‘on the ground’.

## Conclusion

Naturopathy in Australia is currently facing internal and external challenges. Further investigation of significant practice and policy implications of these challenges is critical to understanding the impact that these have on naturopathic healthcare delivery and the naturopathic profession. Given the increasingly mainstream role that naturopaths are playing in the healthcare system in Australia, it is imperative that some of the issues of concern raised by naturopaths receive appropriate policy focus. This may include the development of appropriate regulatory regimes and the development of minimum standards of practice and education that value traditional naturopathic principles and philosophies, as well as ensuring ethical and effective clinical practice.

## Competing interests

The authors declare they have no competing interests.

## Authors’ contributions

JW was involved with conception and design of the study, collection of the data, analysing and interpreting the data and drafting and revising the manuscript; JA was involved with conception and design of the study, analysing and interpreting the data and drafting and revising the manuscript; C-WL was involved in analysing and interpreting the data and drafting and revising the manuscript; AS was involved in analysing and interpreting the data and drafting and revising the manuscript. All authors read and approved the final manuscript.

## Pre-publication history

The pre-publication history for this paper can be accessed here:

http://www.biomedcentral.com/1472-6882/13/15/prepub
